# Time-Based Transition Expectancy in Task Switching: Do We Need to Know the Task to Switch to?

**DOI:** 10.5334/joc.145

**Published:** 2021-03-10

**Authors:** Stefanie Aufschnaiter, Andrea Kiesel, Roland Thomaschke

**Affiliations:** 1 Cognition, Action, and Sustainability Unit, Department of Psychology, University of Freiburg, Freiburg im Breisgau, DE

**Keywords:** Task switching, timing, time-based expectancy, preparation

## Abstract

Recent research has shown that humans are able to implicitly adapt to time-transition contingencies in a task-switching paradigm, indicated by better performance in trials where the task transition (switch vs. repetition) is validly predicted by the pre-target interval compared to trials with invalidly predicted transitions. As participants switched between only two different tasks, not only the transition, but also the specific task was predictable; at least indirectly when taking into account the temporally predicted transition in the current trial together with the task in the previous trial. In order to investigate if the time-based expectancy effect for transition in previous studies was due to a specific task preparation or due to an unspecific transition preparation, three different tasks were used in the present study. One of two possible pre-target intervals (500 and 1500 ms) predicted a task switch in the upcoming trial with 90 % probability, whereas the other interval predicted a task repetition with 90 % probability. Results revealed that participants were able to prepare both upcoming repetition as well as switch requirements based on predictive pre-target intervals. This means that humans seem to be able to prepare a task switch in a rather unspecific manner, most likely by inhibiting the task just performed in the previous trial. By suggesting a two-stage preparation model in which switches as well as repetitions benefit both from time-based transition expectancy, although apparently with different cognitive processes being involved, the present study provides important impulses for future research on the cognitive processes underlying human task-switching behavior.

Every day we engage in multitasking, be it voluntarily or not (see also Himi, Bühner, Schwaighofer, Klapetek, & Hilbert, 2019; Parry & le Roux, 2018). In the laboratory, multitasking is typically investigated by applying either a dual task paradigm, where two tasks have to be processed concurrently (Pashler, 1994), or a task switching paradigm, where two or more tasks have to be executed in succession without temporal overlap (Kiesel et al., 2010; Vandierendonck, Liefooghe, & Verbruggen, 2010; Ward, Hussey, Cunningham, Paul, McWilliams, & Kramer, 2019, see Koch, Poljac, Müller & Kiesel, 2018 for a recent combined discussion of both paradigms). The general finding in task switching studies is that switching to another task takes longer compared to the repetition of the task which has just been executed in the previous trial. This performance difference in reaction times (RTs) between task switches and task repetitions is usually referred to as switch costs (see Kiesel et al., 2010, for a review).

Recently, it has been shown that humans are able to make use of the predictive value of pre-target intervals in order to adjust their anticipatory cognitive control for preparing upcoming task execution requirements (Aufschnaiter, Kiesel, Dreisbach, Wenke, & Thomaschke, 2018a; Aufschnaiter, Kiesel, & Thomaschke, 2018b; Aufschnaiter, Kiesel, & Thomaschke, 2020). Importantly, this enhanced preparation could not only be observed when the upcoming task identity was predicted based on time, but also when only the upcoming task transition (i.e. task switch vs. task repetition) was predicted based on the preceding time interval (Aufschnaiter et al., 2018a). However, as participants switched between only two different tasks in these studies, participants could actually still infer the specific task from the current interval and their knowledge about the previous task. Consequently, by using three tasks instead of two, the present study examines the question whether the time-based expectancy effect for transition is due to specific task preparation, or due to unspecific preparation of task transition.

## Time-Based Task Expectancy in Task Switching

There are several sources of predictability in the task switching paradigm which can be employed in order to adjust anticipatory cognitive control like, for instance, explicit task cues preceding the target stimuli (e.g. Dreisbach, Haider, & Kluwe, 2002; Koch & Allport, 2006) or explicit (Koch, 2005) and implicit (Gotler, Meiran, & Tzelgov, 2003) task sequences. Thus, the predictive value of pre-target intervals is one among other potential sources of task predictability which can be used to enhance preparation for upcoming task requirements.

Task preparation based on duration information was first documented by Aufschnaiter et al. (2018a). They revealed that participants benefit not only from prolonging the pre-target interval (Meiran, 1996; Rogers & Monsell, 1995), but that participants can make use of the predictive value of a pre-target interval in order to adjust anticipatory cognitive control regarding the upcoming task requirements. Most importantly, performance benefits in trials with validly predicted tasks compared to trials with invalidly predicted tasks were independent from the absolute length of the respective pre-target interval, and could be observed in a wide temporal range from 10 ms to even 3000 ms (Aufschnaiter et al., 2020).

The evidence for humans being able to implicitly adapt to time-task contingencies, and to show better performance in trials with validly predicted tasks compared to invalidly predicted tasks, is commonly explained by time-based expectancy as the underlying cognitive process (see Thomaschke & Dreisbach, 2015, for a review). Time-based expectancy is usually investigated by applying the so-called time-event correlation paradigm, a specific variation of the foreperiod paradigm (Schröter, Birngruber, Bratzke, Miller, & Ulrich, 2015), which was introduced by Wagener and Hoffmann (2010). In this paradigm, two pre-target intervals of different absolute length as well as two target stimuli appear equally often, but the combinations of interval and target differ regarding their overall frequency. The short interval is frequently combined with one target, whereas the long interval is frequently combined with the other target. It is assumed that participants learn the contingencies between intervals and targets implicitly by an associative learning mechanism (see Abrahamse, Braem, Notebaert, & Verguts, 2016; Los, Kruijne, & Meeter, 2014; Thomaschke & Dreisbach, 2015). The learned associations between intervals and targets are assumed to generate time-based expectancies which means that expectancy is directed towards the target associated with the short interval at the beginning of a trial. In case the short interval has passed without any target presentation, expectancy is directed towards the target associated with the long interval (see Aufschnaiter et al., 2018a; Thomaschke, Bogon, & Dreisbach, 2018; Volberg & Thomaschke, 2017). Consequently, time-based expectancy is typically indicated by better performance in trials with frequent combinations of interval – target combinations compared to trials with infrequent interval – target combinations.

Please note, that in contrast to time-based expectancy, which means expecting a specific target at a certain point in time, time expectancy refers to as expecting *when* something will happen, independently from *what* exactly will happen at that point in time (Capizzi & Correa, 2018; Grabenhorst, Michalareas, Maloney, & Poeppel, 2019). Time expectancy is typically investigated by applying the above-mentioned foreperiod paradigm where the temporal distance between warning signal and target is manipulated (Los & Agter, 2005; Steinborn & Langner, 2012). However, time expectancy is conceptually different from time-based expectancy, and both forms of temporal expectancy usually do not interact (see Thomaschke & Dreisbach, 2015). For these reasons, time expectancy will not further be discussed in the present study.

## Time-Based Transition Expectancy in Task Switching

Interestingly, previous studies could show that not only preparation for specific task identities is enhanced by the adjustment of anticipatory cognitive control but also the preparation for task transitions (i.e. task switches and task repetitions; Bonnin, Gaonac’h, & Bouquet, 2011; Dreisbach & Haider, 2006, Farooqui & Manly, 2015; Monsell & Mizon, 2006).

For example, Dreisbach and Haider (2006) compared performance in experimental blocks with task switches in 75% of all trials (frequent-switch blocks) with performance in experimental blocks with task repetitions in 75% of all trials (frequent-repetition blocks). Interestingly, the authors found increased RTs for improbable task repetitions in frequent-switch blocks, especially when cues indicated the probability of a task switch before each trial. Moreover, a study by Farooqui and Manly (2015) revealed improved performance in trials with task switches, which had been announced by a subliminal cue at the beginning of a trial. The authors concluded that humans seem to be able to implicitly use subliminal information in order to adjust anticipatory cognitive control processes without being aware of it. Furthermore, it could be shown that participants implicitly make use of the predictive value of pre-target intervals in order to adjust their cognitive control involved in preparatory processes and to show better performance in trials with validly predicted task transitions compared to trials with invalidly predicted task transitions (Aufschnaiter et al., 2018a). In the study by Aufschnaiter et al. (2018a) one of two possible pre-target intervals (500 and 1500 ms) predicted the upcoming task transition (task switch vs. task repetition) with 90% (Experiment 4) probability. Participants showed significantly faster RTs in trials with validly predicted task transitions compared to trials with invalidly predicted task transitions. Importantly, this time-based expectancy of task transition was not restricted to task switches but also task repetitions profited when validly predicted by time. Thus, results support the prevailing view in cognitive research on task switching that task repetitions require cognitive control processes as well (see Altmann, 2004; Dreisbach & Haider, 2006; Koch, 2001).

However, there are two different possible explanations for the time-based expectancy effect for task transition in the experiments by Aufschnaiter and colleagues (2018a). On the one hand, the requirements of the specific task to be executed in the upcoming trial could have been either solely inferred from the pre-target interval being predictive about the upcoming task transition (direct unspecific preparation of task transition). On the other hand, it is also possible that the requirements of the specific task to be executed in the upcoming trial were inferred from both the predictive value of the pre-target interval *and* the knowledge about the previous task in trial n-1 (indirect specific task preparation).

Please note that the first account assuming unspecific preparation of a task transition suggests a role of backward inhibition of the task just executed (see Koch, Gade, Schuch, & Philipp, 2010 for a review on the role of inhibition in task switching)^1^: In case participants are indeed able to prepare a task transition in a direct, rather unspecific manner, this would most likely mean that participants inhibit, at the point in time typical for switches, the task which had just been performed in the previous trial.

However, please note further that some studies suggested that participants cannot benefit from inhibiting the improbable task when being presented with probability cues about the task to be performed in the upcoming trial, if no specific information about the upcoming task requirements is provided (see Dreisbach et al., 2002; Mayr & Keele, 2000; but see Chiu & Egner, 2017 for an alternative explanation). Thus, these authors proposed that inhibition of the improbable task is only possible if specific information about the upcoming task requirements is available.

## Research Question

**Figure 1:**
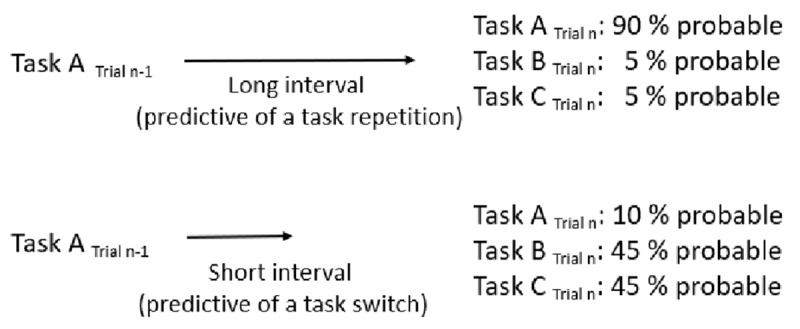
Schematic illustration of probabilities of task A, B and C in trial n when task A has been presented in trial n-1. In the depicted exemplary experimental condition, the long pre-target interval predicts a task repetition, whereas the short pre-target interval predicts a task switch.

To examine the question whether the time-based preparation of a task transition is due to a specific task preparation or due to an unspecific preparation of a task transition, a combination of the standard task switching paradigm (cf. Kiesel et al., 2010) and the time-event correlation paradigm (Wagener & Hoffmann, 2010) was used. The procedure involved two different intervals (500 ms and 1500 ms) and three different tasks. One of the two intervals predicted a task switch in the upcoming trial with 90 % probability, the other interval predicted a task repetition with 90 % probability. Concerning time-based expectancy, three hypotheses can be formulated for this design:

1. The design of the present study did not allow to infer specific information about the task requirements of switch trials: After having executed task A in trial n-1, task B as well as task C were equiprobable when a switch was predicted by the pre-target interval in trial n (see ***Figure 1***). Nevertheless, in case participants are indeed able to prepare a task switch in a direct, but rather unspecific manner, and thus are capable of learning the association between interval and task transition, we would expect a significant time-based expectancy effect for task switches as well as for task repetitions. Such an unspecific preparation for a task switch in a paradigm with three tasks would most likely mean that participants inhibit, at the point in time typical for switches, the task which had just been performed in the previous trial (see Koch et al., 2010, for a review; Strobach, Wendt, & Janczyk, 2018).
2. If a task-specific preparation process is required for time-based transition expectancy, a time-based expectancy effect should not be observable for task switches in a paradigm with three different tasks. In case of a predicted task switch, the only information that can be derived is that the task of the previous trial will probably not occur in the next trial and each of the two tasks which have not been presented in the previous trial can occur with equal probability (see ***Figure 1***). Thus, no time-based expectancy effect should emerge for task switches in case time-based transition expectancy implies a task-specific preparation process. However, in this case, a time-based transition expectancy effect should be observable for task repetitions. If the pre-target interval is predictive for a task repetition, the specific preparation of the upcoming task should be possible, as the task which has been performed in the previous trial can be specifically expected and is most likely to occur at that point in time (see ***Figure 1***).
3. However, it can also be the case that no effect at all can be found neither for task repetitions, nor for task switches, because a *specific* preparation of a task set would only be possible in half of the trials (when time predicts an upcoming task repetition) in the present design. Thus, in case time-based transition expectancy implies a task-specific preparation, this time-based task-specific preparation would only be possible in 50 % of all trials (as in trials with a predicted task switch, each of the two tasks which have not been presented in the previous trial can occur with equal probability and thus no task-specific preparation is possible; see ***Figure 1***). Until now, no study has investigated if time-based expectancy is possible at all if only one of two intervals has a predictive value. Thus, it might be possible that participants do not learn the time-transition contingencies in this case or at least cannot make use of it.

**Figure 2:**
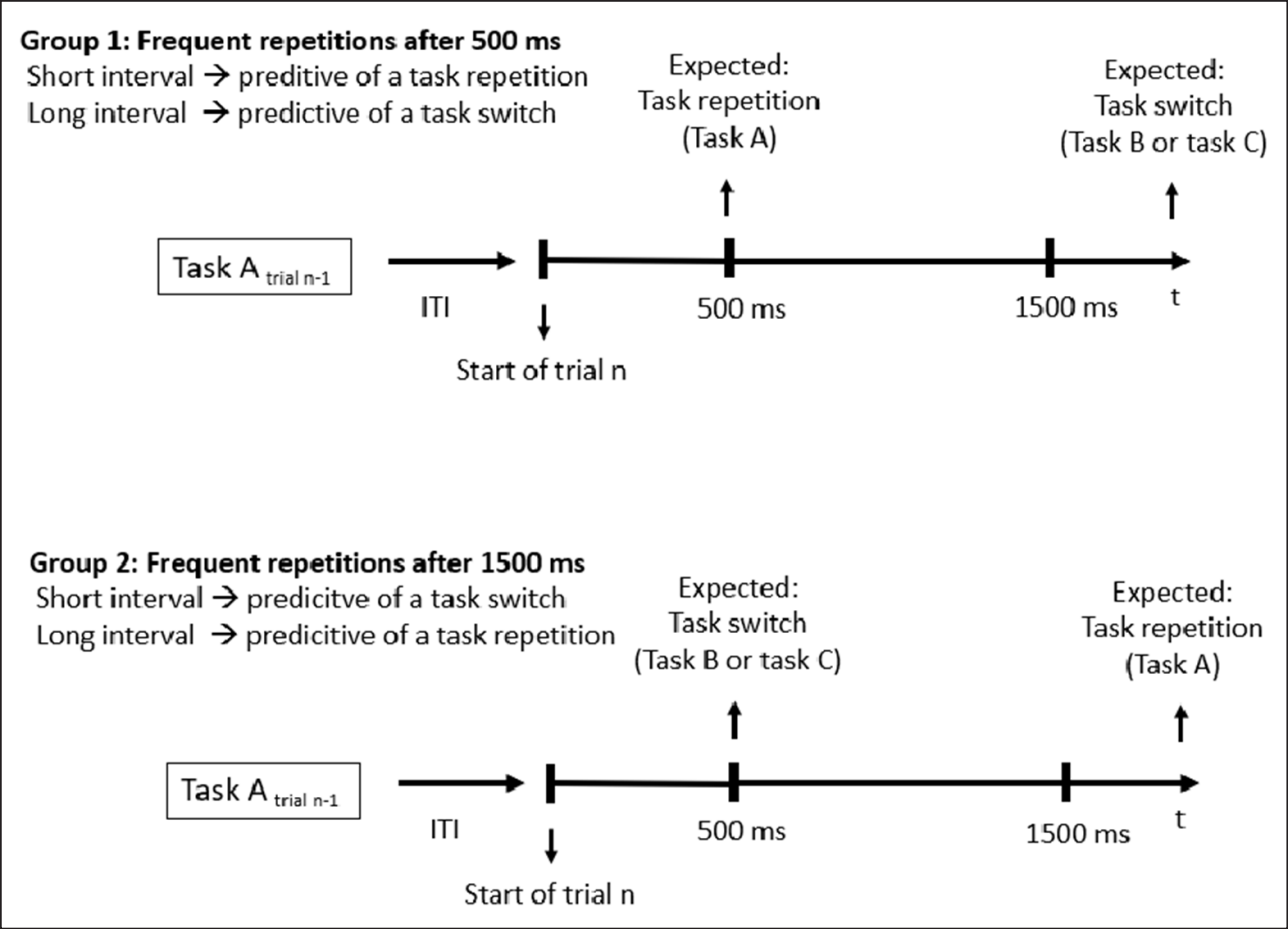
Schematic illustration of the experimental between-subject condition “interval of expected repetition”: Whereas one half of participants (group 1) expected a task repetition to occur frequently after 500 ms in trial n, the other half of participants (group 2) expected a task repetition to occur frequently after 1500 ms trial n.

As could be derived from the hypotheses, we foremost wanted to investigate, if participants are able to expect both, task switches as well as task repetitions, based on predictive intervals. Therefore, we included the within-subject factors “predictability of interval-transition combination” and “task transition” in our analysis. If we would find a main effect for predictability of interval-transition combination and no interaction between predictability of interval-transition combination and transition, this would probably mean that hypothesis 1 would be supported by our data. However, in this case, we assumed inhibitory mechanisms to be involved in time-based expectancy for task switches, as no specific knowledge can be inferred about the task to be performed in the upcoming trial whenever a task switch is validly predicted based on time in a paradigm with three different tasks (see explanation of hypothesis 1 for detailed information). For this reason, we also included the between-subject factor “interval of expected repetition” in our analysis^2^: Whereas half of the participants expected a task repetition to occur frequently after 500 ms (group 1), the other half of the participants expected a task repetition to occur frequently after 1500 ms (group 2; see ***Figure 2***). If humans are able to expect a task switch based on time in a rather generic manner without specific knowledge about the upcoming task, hypothesis 1 predicts that this would most likely mean that inhibitory processes might be involved. Thus, if inhibitory mechanisms are involved whenever a task switch is expected to occur after 500 ms, participants in group 2 should inhibit the task, which had just been performed in the previous trial, during the first 500 ms of the current trial. However, if no stimulus is presented after 500 ms, which means that a task repetition is now to be expected based on the long interval, participants in this group would have to re-activate the task, which had just been inhibited (see ***Figure 2***) and should thus show a smaller time-based expectancy effect for task repetitions compared to participants in group 1, who expected a task repetition to occur after 500 ms.

As our hypotheses also include potential null effects, we conducted a Bayesian ANOVA with default prior scales using JASP (JASP team, version 0.8.1.2) and looked at the Bayes tests for both the main effect of predictability of interval-transition combination (hypotheses 1 and 3) as well as for an interaction between transition and predictability of interval-transition combination (hypothesis 2).

## Method

### Participants

192 participants (66 females^3^; mean age = 29.99, *SD* = 10.41, range = 18–69 years^4^; 162 right-handed^5^) took part in the experiment. Participants were recruited via the online participant recruitment tool *Prolific* (*https://www.prolific.co/*) and took part in exchange for monetary compensation. All participants spoke fluently English, had normal or corrected-to-normal vision, and were naïve concerning the hypotheses. Participants provided written informed consent prior to the experiment. In order to make sure that participants fully concentrated on the experiment without any interruptions and followed the instruction to react as fast and as correctly as possible, we a priori excluded participants who showed an error rate above 25% and who took longer than one hour to complete a session. We suspected these participants of not having fully concentrated on the experiment without any interruptions or distractions and re-collected data for the respecting experimental conditions.

We used a stopping rule to determine the necessary sample size, and decided to stop data collection whenever the Bayes Factor (BF) would get larger than 3 either in favor of the null hypothesis (H0) regarding the main effect of predictability (no difference of mean RTs between trials with predictable interval – transition combinations and trials with unpredictable interval-transition combinations), or in favor of the alternative hypothesis (H1). As the interaction between transition and predictability of interval-transition combination played a crucial role concerning our hypotheses, a further criterion for stopping data collection was that, in addition, the BF regarding the interaction of transition and predictability of interval-transition combination had to get larger than 3 either in favor of H0 (no difference of mean RTs between switch and repetition trials regarding the predictability of interval-transition combinations), or in favor of H1. The Bayesian approach is a model selection procedure that indicates the likelihood ratio of two or more hypotheses based on the given data. Thus, Bayesian analysis provides the possibility of evaluating evidence in favor of the (null-) hypothesis. In this context, a BF in the range of 3 to 10 indicates moderate evidence in favor of the H0 resp. H1 (Lee & Wagenmakers, 2013). For reasons of counterbalancing, we first conducted the respective Bayes analyses after 64 participants, then after 128 participants and finally reached the stopping criteria after 192 participants.

### Apparatus and Stimuli

Participants responded by key presses on the keyboard of their computer (keys w and o; it was not allowed to use the mobile phone or a tablet in order to be eligible to take part in the study). Target stimuli were blue, green or red numbers between 1 and 9, except 5, presented against a black background (Arial typeface, font size 1.5 cm). The fixation cross was the “+” symbol (Arial typeface, font size 1.5 cm). All stimuli were presented centrally on the screen.

### Procedure

Each trial started with a blank screen for 300 ms (inter-trial interval, ITI), which was followed by the presentation of a white fixation cross for a variable interval of either 500 ms or 1500 ms. After this interval, the target stimulus was presented. The order of stimuli was randomized, and each stimulus occurred with equal probability. Depending on the color of the target stimulus (blue, green or red), participants had to judge whether the displayed number was smaller or larger than 5, whether it was odd or even, or whether the displayed number was distant to 5 (e.g. 1, 2, 8, 9) or near to 5 (e.g. 3, 4, 6, 7) on a mental number line. The latter task will be referred to as the inner-outer task from here on. The mapping of colors to tasks was held constant across participants. If the number was displayed in blue, participants had to perform the magnitude judgment task, and if the number was displayed in green, participants had to perform the parity judgment task. A red color indicated the inner-outer task. Responses were given with the same two buttons for all three tasks. The mapping of responses to keys was counterbalanced across participants. Participants were instructed to respond as fast and as correctly as possible. After the detection of an error, the word *Error* was displayed in white on a black screen for 1500 ms. After correct responses, no explicit feedback was given. The duration of the interval predicted the upcoming task transition in the current trial. This means, a task switch occurred frequently after one interval, while a task repetition appeared frequently after the other interval. This means that when the interval was predictive of a task repetition after having executed task A, task A was predictable with 90% probability, whereas task B and task C were each predictable with 5% probability. In contrast, when the interval was predictive of a task switch after having executed task A, task A was predictable with only 10% probability, whereas task B and Task C were each predictable with 45% probability (see ***Figure 1***). Both, the intervals, as well as the three tasks, appeared with the same overall frequencies. The mapping of task transition (switch vs. repetition) to interval was counterbalanced across participants. Participants were not informed that the intervals had different durations, or that these interval durations were correlated with task transitions.

The experiment consisted of two sessions of about 30 min each, which were tested on consecutive days. Both sessions of the experiment consisted of four blocks each: one learning block, and three test blocks. Each block comprised 120 trials. The only difference between learning blocks and test blocks was that after the detection of an error, the instruction was once again presented in white font color on a black screen for 8000 ms in the learning blocks, before the next trial started with the presentation of the ITI. Between blocks, participants could take a break, which they could terminate themselves by pressing the spacebar. After the second session of the experiment, participants had to complete a short survey in which they were asked if they had noticed any temporal regularities in the experiment.

## Results

Following earlier studies on time-based expectancy, we analyzed only the second session (e.g. Thomaschke & Dreisbach, 2013). Data from the learning blocks, from the first three trials of each test block, as well as trials with number repetitions, and trials following an error trial were excluded from analyses. In addition, we excluded trials with RTs < 100 ms from analyses and we removed trials, in which RTs deviated more than 3 standard deviations from the corresponding cell mean, computed separately for each participant, block and experimental condition (Bush, Hess, & Wolford, 1993). Furthermore, trials with errors were removed from the RT analyses.

Based on the sample of 192 participants, we conducted an outlier analyses and excluded three participants from the following analyses due to high error rates in comparison to the other participants (mean percentage of errors for the three excluded participants: 9.2 %, 9.8 % and 15.8 %; mean percentage of errors for all participants: 2.40 %). This resulted in a final sample of 189 participants.

First, we conducted a three-factor ANOVA with the within-subjects factors transition (switch vs. repetition), predictability of interval – transition combination (predictable vs. unpredictable) and the between-subjects factor “interval of expected repetition”, meaning that we took into account if a task repetition was expected after the short or after the long pre-target interval, dependent of the respective experimental condition (for a detailed description of this analysis in theoretical terms, see Introduction). Additionally, we performed Bayesian ANOVA with default prior scales using JASP. Please notice that during the following analyses, we will explicitly mention whenever BF and *p*-value do not support each other, and that we will further indicate explicitly, whenever there was only anecdotal Bayesian evidence in favor of H0 or H1 (in accordance to Lee & Wagenmakers, 2013).

**Figure 3:**
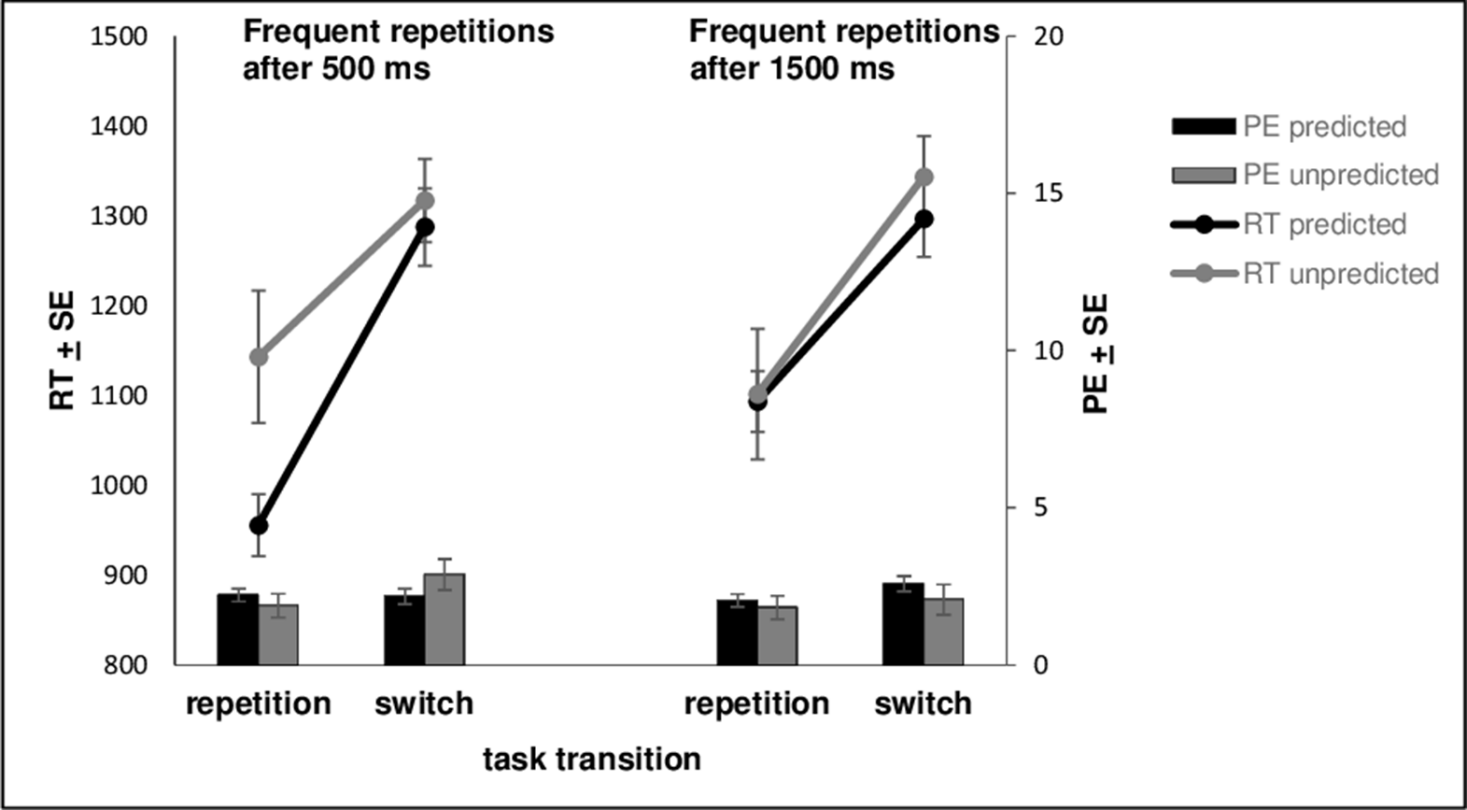
Mean reaction times (RTs in ms; lines) and percentages of errors (PEs in %; bars), depending on predictability of interval – transition combination, are displayed separately for task transition and the between-subject condition “interval of expected task repetition” (500 ms vs. 1500 ms). Error bars represent 1 standard error of the mean.

RTs and error rates are displayed in ***Figure 3***. Results revealed a significant main effect for transition, meaning that responses were faster in trials with task repetitions (*M* = 1073 ms, *SD* = 477) compared to task switches (*M* = 1311^6^, *SD* = 417), *F* (1, 187) = 83.476, *p* < .001, η_p_^2^ = .309. Moreover, the main effect for predictability of interval-transition combination gained significance, *F* (1, 187) = 12.891, *p* < .001, η_p_^2^ = .064, meaning that responses in trials with predictable interval-transition combinations were faster (*M* = 1158 ms, *SD* = 359) than in trials with unpredictable interval-transition combinations (*M* = 1226 ms, *SD* = 492), BF in favor of H1 = 4.159. Whereas the interaction between transition and “interval of expected repetition” did not turn significant, *F* (1, 187) < 1, *p* = .559, η_p_^2^ = .002, BF in favor of H0 = 7.220, the interaction between predictability of interval-transition combination and “interval of expected repetition” turned significant, *F* (1, 187) = 4.555, *p* = .034, η_p_^2^ = .024, BF in favor of H1 = 0.450 (please note that *p*-value and BF did not support each other and that we will not further interpret this interaction in the general discussion for this reason). The interaction between transition and predictability of interval-transition was not significant, *F* (1, 187) = 1.460, *p* = .229, η_p_^2^ = .008, BF in favor of H0 = 3.453, but, however, the interaction between “interval of expected repetition”, transition and predictability of interval-transition combination turned significant, *F* (1, 187) = 3.950, *p* = .048, η_p_^2^ = .021, BF in favor of H1 = 1.982 (anecdotal evidence, see Lee & Wagenmakers, 2013). The main effect of the between-subjects factor “interval of expected repetition” did not turn significant, *F* (1, 187) < 1, *p* = .582, η_p_^2^ = .002, BF in favor of H0 = 4.623.

Given the significant three-way interaction for RTs, we conducted two ANOVAs with the within-subject factor predictability of interval-transition combination (predictable vs. unpredictable) and the between-subject factor interval of expected repetition (expected repetitions after 500 ms, group 1, vs. expected repetitions after 1500 ms, group 2; see also ***Figure 2***) separately for task switches and for task repetitions.

For task switches, we observed a significant main effect for predictability of interval-transition combination, *F* (1, 187) = 6.077, *p* = .015, η_p_^2^ = .031, BF in favor of H1 = 2.108 (anecdotal evidence, see Lee & Wagenmakers, 2013), meaning that responses in trials with predictable interval-transition combinations were significantly faster (*M* = 1292 ms, *SD* = 414) compared to responses in trials with unpredictable interval-transition combinations (*M* = 1330 ms, *SD* = 446). The main effect of “interval of expected repetition”, *F* (1, 187) < 1, *p* = .771, η_p_^2^ = .000, BF in favor of H0 = 2.540 (anecdotal evidence, see Lee & Wagenmakers, 2013), as well as the interaction between predictability of interval-transition combination and “interval of expected repetition” did not turn significant, *F* (1, 187) < 1, *p* = .578, η_p_^2^ = .002, BF in favor of H0 = 5.525. In group 1 (expected repetitions after 500 ms), responses were faster in trials with predictable interval-transition combinations (*M* = 1287 ms, *SD* = 591) compared to trials with unpredictable combinations of interval-transition combinations (*M* = 1317 ms, *SD* = 635) and this was also the case for participants in group 2 (expected repetitions after 1500 ms; *M* = 1296 ms, *SD* = 581 464, in trials with predictable interval-transition combinations vs. *M* = 1343 ms, *SD* = 625 464, in trials with unpredictable interval-transition combinations).

For task repetitions, we also observed a significant main effect for predictability of interval-transition combination, *F* (1, 187) = 5.642, *p* = .019, η_p_^2^ = .029, BF in favor of H1 = 1.421 (anecdotal evidence, see Lee & Wagenmakers, 2013), meaning that responses in trials with predictable interval-transition combinations were significantly faster (*M* = 1024 ms, *SD* = 331) compared to responses in trials with unpredictable interval-transition combinations (*M* = 1122 ms, *SD* = 711). The main effect of “interval of expected repetition”, *F* (1, 187) < 1, *p* = .489, η_p_^2^ = .003, BF in favor of H0 = 4.418, did not turn significant. However, the interaction between predictability of interval-transition combination and “interval of expected repetition” turned significant, *F* (1, 187) = 4.720, *p* = .031, η_p_^2^ = .025, BF in favor of H1 = 1.348 (anecdotal evidence, see Lee & Wagenmakers, 2013). In group 1 (expected repetitions after 500 ms), responses were faster in trials with predictable interval-transition combinations (*M* = 955 ms, *SD* = 471) compared to trials with unpredictable interval-transition combinations (*M* = 1143 ms, *SD* = 1014), *t*(92) = –.2.285, *p* = .025. However, for participants in group 2 (expected repetitions after 1500 ms), this difference in RTs was much smaller (*M* = 1093 ms, *SD* = 464, in trials with predictable interval-transition combinations vs. *M* = 1102 ms, *SD* = 998, in trials with unpredictable interval-transition combinations), *t*(95) = –.508, *p* = .613.

For error rates, results revealed no significant main effects or interactions for the ANOVA with the within-subjects factors transition and predictability of interval-transition combination and the between-subjects factor “interval of expected repetition” (max. *F* = 3.560, min. *p* = 0.61).

Only three of the 192 participants noticed temporal regularities during the course of the experiment, as they stated in the survey after the second session that sometimes, numbers occurred more quickly than others, and hence facilitated expectations for the same color to appear again.

## General Discussion

The present study investigated whether the benefit from time-based transition expectancy in task switching (cf. Aufschnaiter et al., 2018a) is due to an unspecific preparation of a task transition or rather due to a specific task preparation by inferring task identity based on the previous task identity and the prediction of the task transition in trials with validly predicted switches, when only two different tasks are used (cf. Aufschnaiter et al., 2018a). For this purpose, the present study employed three different tasks instead of two (cf. Aufschnaiter et al., 2018a). One of two possible intervals (500 vs. 1500 ms) predicted a task switch in the upcoming trial with 90 % probability, the other interval predicted a task repetition with 90 % probability.

Our results revealed a significant time-based expectancy effect, which was further supported by Bayesian analysis, and importantly, this effect did not interact with task transition (switch vs. repetition). Thus, the assumption of unspecific preparation of a task transition seems to be supported by the present data: As the design of the present study did not allow to infer specific information about the task requirements of switch trials, our results indicate at first glance that participants seem to be able to learn the association between the pre-target interval and task transition and thus seem to prepare a task switch in a direct, but rather unspecific manner.

However, please note that we found a significant three-way interaction between transition, predictability of interval-transition combination and interval of expected repetition (i.e. group), which was supported by at least anecdotal Bayesian evidence. The between-subject factor group meant that we took into account, if participants belonged to the group, which expected task repetitions to occur frequently after 500 ms (group 1), or if they belonged to the group, which expected task repetitions to occur frequently after the long interval (group 2). Importantly, when solely investigating task switches based on the significant three-way interaction, we found a significant predictability effect, which was, though, supported by at least anecdotal evidence given our Bayesian analysis. However, the predictability effect for task switches did not interact with group, which was further supported by Bayesian analyses. This means that based on these additional analyses we can infer that task switches really seem to benefit from time-based predictability and that participants seem to be able to prepare a task switch in a rather unspecific manner without knowledge about the specific requirements of the upcoming task.

Assuming that participants benefitted from inferring specific knowledge about the task to be performed in the upcoming trial when a task repetition was validly predicted based on duration information (see also Koch, 2008), the question remains, though, which cognitive preparatory mechanism supports switch preparation based on time. As it was already stated above, an unspecific preparation for a task switch in a paradigm with three tasks would most likely mean that participants inhibit, at the point in time typical for switches, the task that had just been performed in the previous trial (see Koch et al., 2010, for a review). And importantly, this is exactly what our data suggest: When only investigating task repetitions based on the significant three-way interaction, we find a significant interaction between predictability of interval-transition combination and group, for which the corresponding Bayesian analysis revealed at least anecdotal evidence. In group 1, where participants expected a task repetition to occur frequently after the short interval of 500 ms, we find a time-based expectancy effect for task repetitions, whereas in group 2, where participants expected task repetitions to occur frequently after the long interval of 1500 ms, no time-based expectancy effect for task transitions could be observed. While task switches seem to benefit from time-based expectancy in both groups of participants (see above), participants obviously could only prepare for a task repetition based on a predictive interval, when belonging to group 1, where they expected task repetitions to occur frequently after the short interval of 500 ms, explaining the significant three-way interaction. However, as already stated above, this finding, which may come unexpected at first sight, provides first evidence for the account of inhibitory processes underlying time-based expectancy for task transition.

Let us assume that the task, which had just been performed, is inhibited when a switch is predicted based on time after the short interval of 500 ms. This means that participants, who expect a switch to occur frequently after 500 ms should inhibit the task just performed in the previous trial during the first 500 ms of the current trial. When no stimulus appears after 500 ms, they have to re-direct their expectancy in the direction of a task repetition, which is likely to appear after the long interval of 1500 ms. However, this would mean that they would have to “re-activate” the inhibited task, which should imply a time-consuming cognitive effort. And indeed, as it was explained above, for participants who expected task switches to occur after 500 ms, we observed, even numerically, no expectancy effect when a task repetition occurred after 1500 ms (even if the appearance of a task repetition after 1500 ms implied a predictable interval-transition combination and should thus lead to performance benefits). This observation speaks in favor of inhibitory processes being involved in time-based expectancy for task switches, which cannot be easily overcome if expectancy has to be re-directed from an expected task switch towards an expected task repetition.

Though, previous studies suggested that the requirement for inhibition is that the upcoming task can be specifically prepared, be it a task switch or a task repetition (see Dreisbach et al., 2002). However, in the present study, a task could only be specifically prepared if the interval predicted a task repetition: In this case, the task which had been just performed in the previous trial was likely to occur in the following trial with 90% probability. As our results also revealed a time-based expectancy effect for task switches, where no explicit information about the specific task requirements of the upcoming switch trial could be derived, the present study questions previous studies which stated that inhibition processes require the possibility of specific task preparation, and rather suggests an unspecific switch preparation whenever a task switch is validly predicted based on time.

Based on previous studies, suggesting cognitive preparatory processes, which take place both in repetition and switch trials (see Dreisbach et al., 2002; Koch, 2005), we suggest a two-stage preparation model in which switches as well as repetitions benefit both from time-based transition expectancy. In this context, two-stage models of task set reconfiguration in switch trials have been proposed by numerous studies (see Kiesel et al, 2010; Koch et al., 2018): The first stage (the so-called “advance reconfiguration”, Rogers & Monsell, 1995; “goal shifting”, Rubinstein, Meyer, & Evans, 2001) contributes to preparation for the upcoming cognitive requirements before target presentation. The second stage (“rule activation”, Koch et al., 2018) can only be passed if the specific target stimulus is provided. Although two-stage models are usually discussed in the context of upcoming switch requirements, we propose that two different preparatory stages might also play a role regarding time-based expectancy of task repetitions: Whenever a task repetition is validly predicted by time, the first stage might involve a specific task preparation, by inferring knowledge about the identity of the task just performed in the previous trial (as the explicit task cue, the color, is not presented until target onset). The possibility for specific task preparation during the first stage of the preparation process might account for the at least numerically larger expectancy effect for task repetitions after 500 ms in group 1, where task repetitions were expected to occur frequently after 500 ms, compared to task switches after 500 ms in group 2, where task switches were expected to occur frequently after 500 ms (see ***Figures 2*** and ***3***). Moreover, our results suggest that participants are able to prepare also task switches based on a predictive pre-target interval and thus, also the first preparation stage before target onset in switch trials seems to benefit from time-based expectancy. Based on our conclusions, on which we elaborated above, we suggest that in case a task switch is validly predicted based on time, the first preparation stage for expected task switches might involve the inhibition of the task just performed and might allow to implement an unspecific switch readiness.

Furthermore, regarding future studies, our results offer implications for studies in the context of hierarchical task switching (see Schneider & Logan, 2006). Future studies should, for example, investigate, if participants can benefit from time-based transition expectancy, when an interval is not only predictive of the requirement to switch on a single trial basis (as it was the case in the present study) but when it is predictive of the frequency of switches in a whole task sequence. In this context, a recent study by Chiu and Egner (2017) seems to provide important implications regarding future research on time-based transition expectancy in task switching: The authors found that humans are able to associate certain stimuli with increased switch requirements and show reduced switch costs when being presented with these stimuli. Most importantly, by employing three different tasks (as it was the case in the present study) the authors assume that a general switch readiness as a cognitive bottom-up process is possible, which has nothing to do with the preparedness to switch to an alternative task, but rather with a state of increased cognitive flexibility. As in the study of Chiu and Egner (2017) switch readiness was induced on the level of single trials, indicated by item-specific switch probability (ISSP) effects, it would indeed be interesting to induce switch readiness for longer task sequences based on predictive intervals in order to investigate if switch readiness can be built up in a rather flexible manner between whole task sequences. Interestingly, a recent study by Liu and Yeung (2019) investigated the influence of explicit instructions about switch requirements in an upcoming task sequence and revealed evidence that it is possible to induce different meta-control states which can be adapted given different probabilities of frequent-switch sequences compared to infrequent-switch sequences. However, please notice that in the study of Liu and Yeung (2019), participants were explicitly instructed about switch requirements in an upcoming task sequence. To our knowledge, no study has ever investigated before if it is possible to prime a global switch readiness regarding upcoming task execution requirements in whole sequences in an implicit manner (as it would be the case when predicting the switch likelihood in upcoming task sequences based on predictive intervals). Interestingly, the fact that previous studies could show that participants are able to use contextual information to prepare for an increase of upcoming task switches and that they maintain this information across many trials indicates a sort of higher-level cognitive control (see Braver, Reynolds, & Donaldson, 2003; Dreisbach & Haider, 2006), which may speak in favor of the possibility of inducing time-based transition expectancy on a higher-order sequence level.

Taken together, the present results revealed evidence that humans are able to prepare a task switch in a rather unspecific manner whenever a task switch is validly predicted by the pre-target interval, most likely by inhibiting the task just performed in the previous trial. Moreover, a time-based expectancy effect was also observed for task repetitions in the present study, probably by inferring specific knowledge about the identity of the task just performed in the previous trial when a task repetition was validly predicted based on time. We suggest a two-stage preparation model in which switches as well as repetitions benefit both from time-based transition expectancy, although apparently with different cognitive processes being involved. Furthermore, by demonstrating that humans seem to be able to prepare for task switches in a direct, unspecific way without being presented with specific knowledge about the upcoming task requirements, the present results stimulate the highly topical research field of task switching by challenging future research on the cognitive processes underlying human task switching behavior and additionally provoke relevant impulses regarding future investigations of time-based expectancy.

## Data Accessibility Statement

Raw data of the reported experiment are available via the Open Science Framework, DOI: *10.17605/OSF.IO/Q7RT2*.

## Footnotes

Backward inhibition in task switching has been majorly investigated by employing three different tasks. For example, Mayr and Keele (2000) found N-2 task repetition costs which were indicated by slower responses in the last trial of an ABA sequence compared to the last trial in a CBA sequence. The authors explained these N-2 task repetition costs in the former case, which they called backward inhibition effect, with the inhibition of task A once this task had been executed and is actively suppressed in order to perform task B. This inhibition of task A after having executed the N-2 trial leads to worse performance once this task has to be performed again in an ABA sequence (however, see Grange, Kowalczyk, & O’Loughlin, 2017, for an alternative explanation).

Considering the “interval of expected repetition” seems also interesting because of the following reason: In case of a short/long pre-target interval, we simultaneously introduce a variation of the response-to-stimulus interval (RSI) between trials, i.e. the respective RSI is short or long. Given that previous studies indicated that RSI manipulations impact on switch costs (e.g. Meiran, Chorev, & Sapir, 2000), mainly by affecting performance in task repetitions (Horoufchin, Philipp, & Koch, 2011), it seems reasonably to consider types of expectations for short/long intervals separately.

One participant indicated “other”.

One participant did not indicate his/her age.

Six participants indicated that they were ambidextrous.

Please note that we will refer to the estimated means for each condition in the whole results section.
